# Critical reappraisal of anorectal function tests in patients with faecal incontinence who have failed conservative treatment

**DOI:** 10.1007/s00384-012-1415-9

**Published:** 2012-02-18

**Authors:** T. J. Lam, C. J. J. Mulder, R. J. F. Felt-Bersma

**Affiliations:** Department of Gastroenterology and Hepatology, VU University Medical Center, P.O. Box 7057, 1007 MB Amsterdam, The Netherlands

**Keywords:** Faecal incontinence, Diagnostic tests, Anorectal manometry, Anal endosonography, Anal sphincter

## Abstract

**Objective:**

Anorectal function tests are often performed in patients with faecal incontinence who have failed conservative treatment. This study was aimed to establish the additive value of performing anorectal function tests in these patients in selecting them for surgery.

**Patients and methods:**

Between 2003 and 2009, all referred patients with faecal incontinence were assessed by a questionnaire, anorectal manometry and anal endosonography. Patients with diarrhea, inflammatory bowel disease, pouches or rectal carcinoma were excluded.

**Results:**

In total, 218 patients were evaluated. Of these, 107 (49%) patients had no sphincter defects, 71 (33%) had small defects and 40 (18%) had large defects. Anorectal manometry could not differentiate between patients with and without sphincter defects. Patients with sphincter defects were only found to have a significantly shorter sphincter length and reduced rectal capacity compared to patients without sphincter defects. Forty-three patients (20%) had a normal anal pressures ≥40 mmHg. Seventeen patients (8%) had also a dyssynergic pelvic floor both on clinical examination and anorectal manometry. Fifteen patients (7%) had a reduced rectal capacity between 65 and 100 ml. There was no difference in anal pressures or the presence of sphincter defects in these patients compared to patients with a rectal capacity >150 ml. There was no correlation between anorectal manometry, endosonography and faecal incontinence severity scores.

**Conclusion:**

In patients with faecal incontinence who have failed conservative treatment, only anal endosonography can reveal sphincter defects. Anorectal manometry should be reserved for patients eligible for surgery to exclude those with suspected dyssynergic floor or reduced rectal capacity.

## Introduction

Faecal incontinence (FI) is a disabling condition with an estimated prevalence of 6% in the adult population [1]. Causes of FI are disruption of the anal sphincters secondary to obstetric or surgical trauma, neurologic impairment related to nervus pudendus damage due to chronic straining during delivery or chronic constipation, neuropathy such as diabetes mellitus and multiple sclerosis, decreased rectal capacity (RC) secondary to inflammatory bowel disease, radiation proctitis or irritable bowel syndrome (IBS), diarrhea, reduced mental awareness and the physical inability to reach toilet facilities.

Treatment options for FI are limited. The first step in the management of patients with FI is often dietary modification with fibre and physiotherapy or biofeedback [2–4]. The efficacy of these therapies varies from 50% and 70% for dietary fibre and biofeedback, respectively [5–7]. In patients who are refractory to conservative treatment, surgical intervention aimed at correcting a diagnosed sphincter defect, would be the next approach. Other surgical techniques such as sacral nerve stimulation (SNS), gracilis plasty and artificial bowel sphincter creation may be offered to selected patients and are only performed in limited centers [8, 9].

Anal endosonography and MRI are considered as valuable tools for demonstrating the integrity of the anal sphincters and consequently play a key role in helping to select patients for surgical repair [10, 11]. The sensitivity and specificity of anal endosonography reach almost 100% in identifying anal sphincter defects [12, 13]. Moreover, the reproducibility for sphincter defects and anal sphincter thickness is excellent [14]. Furthermore, the presence of atrophy of the sphincters can be established [15, 16].

Anorectal manometry is used to measure the anal pressures objectively and to determine the RC. Anal manometry can demonstrate lower sphincter pressures in patients with anal sphincter defects, but also in patients with atrophy and is not discriminatory [15, 16]. Anal manometry cannot predict the efficacy of physiotherapy and biofeedback [17].

Considering these diagnostic and therapeutic options, the key question is which tests influence clinical decision making. Fibres are a first line, easy to implement, inexpensive treatment and the effect becomes clear after several weeks. Physiotherapy or biofeedback involves more effort from both patient and physiotherapist and is more costly. Anorectal function testing cannot predict the success of physiotherapy or biofeedback [17]. In patients who are eligible for surgery, demonstration of a large sphincter defect is important. Contraindications for sphincter repair are other (concomitant) causes such as diarrhea, severe atrophy of the sphincters, dyssynergic pelvic floor and a very small rectal compliance. Moreover, for other surgical options like SNS, gracilis plasty and artificial bowel sphincter creation, diarrhea, dyssynergic pelvic floor and a small rectal compliance are contraindications.

Although it is well known that anorectal manometry cannot identify anal sphincter defects, it is still routinely performed and many investigators believe it is important or equally important as anal endosonography and contributes greatly to its test results. The aims of this study were to evaluate whether the addition of anorectal manometry to anal endosonography provided additional information to guide the surgical management of patients with FI who have failed conservative treatment and to establish clear recommendations for the targeted use of these tests.

## Materials and methods

### Study population

All consecutive patients referred for the evaluation of FI to our function laboratory between 2003 and 2009 were included. They were prospectively assessed by a comprehensive questionnaire regarding their perianal complaints, including the duration of symptoms, frequency of bowel movements, stool consistency and the use of pads. Other lines of enquiry included the presence of urinary incontinence, the use of medications and the social impact of FI on their lifestyle. Furthermore, a comprehensive past obstetric, surgical, and drug history was taken. The severity of incontinence was graded using the Vaizey and Wexner scores [18, 19].

Patients with inflammatory bowel disease, proctitis, pouches or rectal and prostate carcinomas were excluded. Since diarrhea by itself can cause FI, patients with chronic diarrhea were also excluded. Diarrhea was considered as a stool frequency of more than three times a day and the loss of liquid stools. For the consistency of the stool, a nominal scale (liquid, soft, solid and variable) was used.

We classified the patients into three groups: (1) those without anal sphincter defects; (2) those with small sphincter defects; and (3) those with large sphincter defects. A defect comprised at least 30 degrees of the circumference of the sphincter. A sphincter defect was considered small if the circumference was less than 25% and large if it was more than 25%.

### Anorectal manometry

A four-microtip transducer, water-perfused catheter (Mui Scientific Type SR4B-5-0-0-0, Mississauga, Ontario, Canada) was used. The water-perfusion method was performed by means of a pull-through technique. With the patient lying in the left lateral position, the catheter was introduced into the rectum. After introduction into the rectum, the catheter was withdrawn with the automatic puller at a speed of 1 mm/s. The maximum basal pressure (MBP) was measured as the mean of highest pressure in rest (normal 40–80 mmHg). The sphincter length (SL) was equivalent to the length over which the MBP was measured. The maximum squeeze pressure (MSP) was measured as the mean increase of pressure from basal pressure during squeezing (normal ≥40 mmHg). The patient was then asked to strain on three separate occasions. No relaxation on straining was defined as a lack of pressure fall in the anal canal on straining under three consecutive attempts. Paradoxical contraction was defined as an increase of anal basal pressure upon straining.

The rectoanal inhibitory reflex (RAIR) was elicited by inflating the rectal balloon to a position where the MBP was at its highest. The volume necessary for the inhibition and recovery of the MBP was recorded.

The rectal compliance was determined with the rectal balloon. Air was inflated manually sing a syringe at a speed of 60 ml in 15 s. The volume of air needed to be inserted to illicit the first sensation of rectal distension, the urge to defecate and the onset of intolerable distension, which is similar to RC, was measured.

### Anal endosonography

Anal endosonography was performed using a three-dimensional diagnostic ultrasound system (Hawk type 2050; B-K Medical, Naerum, Denmark) with a rotating endoprobe with two crystals, covering 2–16 MHz (focal range 2–4.5 cm) (diameter 1.7 cm), producing a 360° view. During recording, the crystals were automatically pulled back by an internal puller, allowing longitudinal distances to be measured. After the endosonography, images were reconstructed into 3D images using computer software. Further details of the methodology of anal endosonography have been previously published [20]. The aspect of the puborectal muscle, external anal sphincter (ESD), internal anal sphincter and submucosa were described. Defects in the ESD were described as hypoechogenic lesions and the extent of the defect was axially measured in hours. The 12 o’clock position was designated as anterior and the 3 o’clock position as left lateral. The 3 o’clock position was located at 90 degrees rotating clockwise. The length of the defect was indicated as proximal, distal or total. Internal anal sphincter defects (ISD) were described as disruption or irregularity of the hypo-echogenic ring. Atrophy of the EAS was judged upon its reflection of the outer interface (border ESD and sub-adventitial fat), reflection pattern and length [21].

### Statistical analysis

Fisher’s exact test and Pearson chi-square test were used to compare proportions where appropriate. Student’s *t*-test was used to compare continuous data. Spearman correlation coefficients were used to determine relationships between the anal pressures and the Vaizey score and Wexner score. All *P* values were two-tailed and statistical significance was taken as a *P* value of less than 0.05. Analyses were performed with the statistical software SPSS version 15.0.

## Results

### Clinical features

In total, 626 patients underwent anorectal function evaluation. Of them, 218 patients met the inclusion criteria for FI and were included in the study (Table 1). Ten patients (5%) were men. Of the remaining 208 female patients, 192 (92%) had at least one previous vaginal delivery (mean 2.2, range 0–9). The mean duration of FI was 4.9 years.

**Table 1:** Demographic and anorectal manometry measurements of the patients with and without sphincter defects on anal endosonography

	Patients with no defects, *n* = 107(49%)	Patients with small defects, *n* = 71(33%)	Patients with large defects, *n* = 40(18%)
Age (years)	63 (33–90)^¶£^	57 (31–83)^¶^	49 (21–73)^£^
Female (%)	98 (92%)	70 (99%)	40 (100%)
Medical history			
Diabetes mellitus (%)	6 (6)	3 (4)	1 (3)
Multiple sclerosis (%)	4 (4)	0	0
Parkinson’s disease (%)	1 (1)	1 (1)	0
Vascular problems (%)	5 (5)	0	0
Surgical history			
No surgical history (%)	38 (36)^$^#	11 (15)^#^	0^$^
Colorectal/anal (%)	15 (15)	8 (11)	11 (28)
Urogenital (%)	53 (49)	53 (74)	29 (73)
Other^a^ (%)	1 (1)	0	0
Anorectal manometry			
MBP (mmHg)	40 (10–80)^^^	37 (10–70)	35 (10–65)^^^
MSP (mmHg)	30 (5–80)	28 (5–60)	28 (10–80)
SL (cm)	3.1 (1–6)^%^	3.0 (1–5)	2.8 (1–4)^%^
Relaxation			
Yes (%)	98 (93)	63 (89)	37 (97)
No (%)	6 (6)	5 (7)	0
Paradox (%)	2 (2)	3 (4)	1 (3)
RAIR			
Yes (%)	105 (98)	69 (97)	40 (100)
No (%)	2 (2)	2 (3)	0
FS (ml)	82 (15–300)	66 (10–240)	66 (15–200)
Urge (ml)	142 (35–300)	116 (30–250)	117 (50–240)
RC (ml)	209 (70–350)^*&^	172 (65–300)^*^	172 (90–350)^&^

Small defect <25% circumference

Large defect >25% circumference

*MBP* maximum basal pressure, *MSP* maximum squeeze pressure, *SL* sphincter length, *RAIR* rectoanal inhibitory reflex, *FS* first sensation, *RC* rectal capacity

^a^Other: surgery for lumbar disc herniation

^¶^ *P* = 0.001

^£^ *P* < 0.001

^$^ *P* < 0.001

^#^ *P* = 0.004

^^^ *P* = 0.09

^%^ *P* = 0.07

*P < 0.001

^*&*^ *P* = 0.006

### Anorectal manometry

#### Anal pressures

Male patients had a higher MSP (39 vs. 28 mmHg; *P* = 0.03) and longer SL (3.6 vs. 3.0 cm; *P* = 0.04) than female patients (Table 1). Forty-three (20%) patients had normal pressures (MBP and MSP ≥40 mmHg). Fifty (23%) and ten (5%) patients had low pressures and very low pressures (MBP and MSP <40 and <20 mmHg, respectively). Nine patients (8%) could not relax their pelvic floors properly on clinical examination and manometry.

#### Rectal compliance

Fifteen patients (7%) had an RC between 65 and 100 ml. These patients did not differ from patients with an RC >100 or patients with an RC >150 ml with regard to anal pressures and the presence of sphincter defects. There was no difference in RC between patients with normal and (very) low pressures. When analyzing RC parameters for male and female patients separately, the results remained the same.

### Anal endosonography

The frequency and type of sphincter defects are presented in Table 2. Patients without sphincter defects were older, had less previous surgery and had a larger RC in comparison to patients with an anal sphincter defect. Furthermore, patients with large defects tended to have a lower MBP (35 vs. 40 mmHg; *P* = 0.09) and a shorter SL (2.8 vs. 3.1 cm; *P* = 0.07) compared to patients without sphincter defects. Four patients with large sphincter defects had an RC ≤100 ml. No differences were found in anal manometry findings between patients with small and large defects as well as patients with an ESD and combined ISD and ESD.

**Table 2:** Findings of anal endosonography

	Patients with no defects (*n* = 107)	Patients with small defects (*n* = 71)	Patients with large defects (*n* = 40)
Type of sphincter defect			
ESD (%)	n/a	66 (93%)	11 (28%)
ISD (%)		0	0
Combined ISD and ESD (%)		5 (7%)	29 (73%)
Sphincter atrophy (%)	34 (32%)	7 (10%)	4 (10%)

*ESD* external sphincter defect, *ISD* internal sphincter defect, *n/a* not applicable

Patients with atrophy had a lower MBP (34 vs. 39 mmHg; *P* = 0.04) and a lower MSP (23 vs. 30 mmHg; *P* = 0.001) compared to patients without atrophy. In the group of patients without defects, patients with atrophy had a lower MBP (35 vs. 43 mmHg; *P* = 0.01) and a lower MSP (24 vs. 32 mmHg; *P* = 0.004) compared to patients without atrophy.

### Faecal incontinence scores

The mean Vaizey score and the Wexner score were 15 (range 6–22) and 13 (range 6–20), respectively. The Vaizey score did not correlate with the MBP, the MSP or the RC. Similar findings were found for the Wexner score. However, when the population was subdivided with respect to the stool consistency, a correlation was found between the Vaizey score and the MBP and MSP in patients with soft stool (*r* = −0.27, *P* = 0.01 and *r* = −0.23, *P* = 0.03, respectively). Regarding the Wexner score, a significant correlation was found with the MSP in patients with soft stool (r = −0.27, *P* = 0.01). No correlation was found between FI scores and sphincter defects.

## Discussion

After therapeutic failure of dietary fibre and physiotherapy, referral and subsequent investigation of patients with FI generally follows. For the detection of anal sphincter defects, anal endosonography (or MRI) is mandatory and considered as the gold standard. Our study has confirmed that anal manometry has no contribution in detecting anorectal sphincter defects. Although patients with large defects tended to have lower MBP (*P* = 0.09) and shorter SL (*P* = 0.07) than patients without sphincter defects, anorectal manometry could not differentiate between patients with and without defects. Other studies have shown comparable results [13, 22]. Anorectal manometry reflects the anal pressures; low pressures are caused by anal sphincter defects, pudendal neuropathy or both.

A novel anorectal manometry technique, the anal pressure vectography has been developed. It evaluates the radial pressures from a quantified vector symmetry index (VSI) that indicates the anatomical integrity of the anal sphincter. A recent study showed that the VSI was as sensitive as anal endosonography in diagnosing an anal sphincter defect [23]. However, the low pressures measured by VSI cannot differentiate between internal and external sphincter defects and the position of the catheter in the patient is crucial. In contrast with anal endosonography, the internal and external sphincter can be clearly visualized and the location of the sphincter defects is directly related to other anatomical structures. Furthermore, anal endosonography is less time consuming and easier to perform than vectography. Although VSI can produce interesting data for research purposes, it has no place in the work-up of the patient with FI. Anal endosonography is preferable considering the costs and availability.

If anorectal manometry is not useful in the next step of detecting sphincter defects, then what is its contribution in patients who are eligible for surgery? Exclusion of patients with functional abnormalities, such as IBS patients with a small RC or patients with a dyssynergic pelvic floor, seems wise. Attempts to repair, strengthen or replace the anal sphincter are not logical in the presence of a concomitant functional disorder.

Four (10%) of the patients with a large sphincter defect, which means that they have an indication for surgery, had an RC ≤100 ml. Patients with an RC of less than 100 ml are at risk for FI and patients with an RC of less than 60 ml are all incontinent, even in the presence of normal sphincter pressures [24]. In the absence of proctitis, pouches and previous anorectal surgery, IBS is the cause of a relatively low RC. Therefore, RC measurement is important to exclude IBS with a small RC in patients with FI before surgical intervention.

Some patients have a dyssynergic pelvic floor and therefore have a disturbed coordination of their sphincters. These patients are unable to contract their pelvic floors when faeces arrive and some patients consequently become faecally incontinent. In 17 patients (8%), we found a non-relaxing pelvic floor, both on clinical examination and anorectal manometry. Anal manometry can be used to confirm the finding of a hypertonic pelvic floor with clinical examination. Clinical examination by an experienced investigator is just as valuable as anorectal manometry [25] and therefore anorectal manometry for this indication is not necessary. Furthermore, we found in patients with atrophy a lower anal pressure, which conforms with other studies [26, 27].

Does anal manometry have a predictive value with regard to the outcome of sphincter repair or other surgical modalities? The present study showed that anorectal manometry and anal endosonography findings do not correlate well with FI scores. We found a weak correlation between the Vaizey score and the MBP and the Vaizey score and MSP in patients with soft stools. In other recent studies, there was no correlation between the severity of FI and anorectal manometry results observed [28–30]. These findings again underscore the multi-factorial etiology of FI. Since there is no correlation with the severity of FI, little or no predictive value of anorectal manometry in the efficacy of the treatment for FI can be expected [29, 31].

**Fig. 1:**
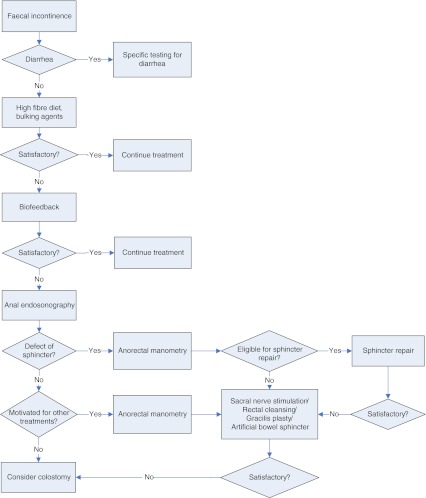
Flow chart of evaluation of patients with faecal incontinence

An algorithm for the treatment of FI is given in Fig. 1. Assessment should begin with a thorough clinical work-up. A detailed medical history and clinical examination with a rectal examination are mandatory. In general, the etiology of FI becomes obvious. Patients with diarrhea or proctitis need further evaluation and targeted treatment. Following this, conservative measures (fibres, physiotherapy) should be applied. In the event of treatment failure, the patient should undergo anal endosonography to demonstrate or exclude a large sphincter defect. In cases of doubt, notwithstanding, patients who are eligible for surgical repair should be evaluated by anorectal manometry, which allows an objective measurement of anal pressures, relaxation of the pelvic floor and RC. Patients who are motivated for other treatments such as SNS, gracilis plasty or artificial bowel sphincter creation, also need be fully evaluated pre-operatively [8, 9, 32, 33]. When all therapies fail, colostomy can be an option.

In conclusion, the present study confirms the importance of anal endosonography in revealing anal sphincter defects and consequently in selecting patients for surgical repair. Although anorectal manometry can provide additional information, it should not be performed routinely in every patient who has failed conservative treatment for FI. Anorectal manometry should be performed in patients selected for surgical intervention to exclude functional abnormalities like dyssynergic floor or IBS with a small RC. In the current adverse economic climate where healthcare budgets are constrained, it is important to reserve the use of tools such as anorectal manometry for those who may benefit from it.
